# Idiopathic Renal Infarction and Anticoagulation

**DOI:** 10.1055/s-0039-1698757

**Published:** 2019-10-09

**Authors:** Maurice I. Khayat, Robert Nee, Dustin J. Little, Stephen W. Olson

**Affiliations:** 1 Department of Nephrology, Walter Reed National Military Medical Center, Bethesda, Maryland, United States

## Introduction

Idiopathic renal infarction (iRI) is rare and the pathophysiology is not well understood. 1 There is no consensus treatment strategy for iRI, because previous studies have not reported long-term outcomes based on therapeutic intervention. 2 3 4 5 6 7 8 9 10 11 12 13 14 15 We sought to determine if anticoagulation or nonanticoagulation was associated with a higher incidence of recurrent arterial thrombosis, de novo venous thrombosis, bleeding event, or development of long-term hypertension, proteinuria, or chronic kidney disease (CKD).

## Methods

**Fig. 1:**
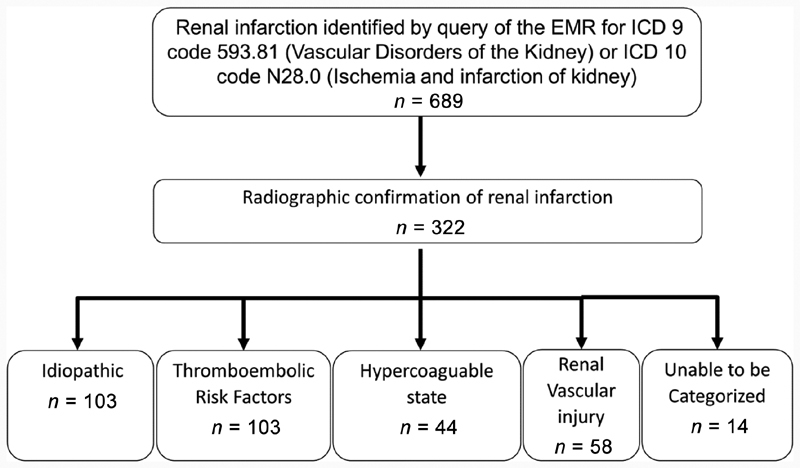
A flow diagram of idiopathic renal infarction case selection.

We conducted a retrospective iRI cohort study to compare long-term outcomes between anticoagulation and nonanticoagulation therapy. We first queried the military electronic medical record (EMR) system, composed of approximately 10 million current and prior service members and their dependents, for the International Classification of Diseases 9 and 10 codes for renal infarction (593.81 and N28.0, respectively). There were 689 potential cases of renal infarction identified. After a full review of the EMR, only 322 were found to have radiologic confirmation of renal infarction. A total of 103 cases were determined to be idiopathic renal infarcts as defined by the absence of known thromboembolic risk factors, hypercoagulability, or vascular pathology etiologies ( Fig. 1 ). A thromboembolic risk factor was defined by diagnostic evidence or documentation by a cardiologist of a patent foramen ovale, ventricular dilatation or aneurysm, valvular vegetations, atrial fibrillation, or significant aorta plaque burden ( *n* = 103). Hypercoagulability was defined by documentation by a hematologist or presence of factor V Leiden, protein C or S deficiency, anti-B2-glycoprotein antibody, anticardiolipin antibody, antiphospholipid antibody, lupus anticoagulant antibody, genetic risk, systemic lupus erythematosus, systemic vasculitis, malignancy, inflammatory bowel disease, pregnancy, or nephrotic syndrome on chart review ( *n* = 44). Renal vascular pathology was defined by documentation in a vascular surgery note or evidence of renal artery stenosis or aneurysm, trauma, or association with abdominal or vascular surgery in close anatomic proximity on chart review ( *n* = 58). Fourteen cases did not have adequate documentation of a negative secondary work-up for iRI assignment. Prespecified background clinical data ( Table 1 ) and primary outcomes were then collected for all 103 iRI cases. Long-term outcomes included recurrent arterial thrombosis by imaging, de novo venous thrombosis by imaging, new or worsening hypertension (two blood pressure readings of >140/90 mm Hg or the addition/escalation of antihypertension mediations), new proteinuria (≥ 1+ on urinalysis in the absence of infection), a bleeding event (confirmed by specialist or emergency department with imaging or procedure), or new stage 3 CKD (estimated glomerular filtration rate less than 60 mL/min/1.73 m ^2^ by CKD-Epi Creatinine equation) after iRI diagnosis. Initial comparison groups were anticoagulation ( *n* = 47) and nonanticoagulation treatment. The nonanticoagulation group ( *n* = 56) was further subdivided into an antiplatelet group ( *n* = 37) and a group with either no treatment or undocumented over-the-counter aspirin (ASA) treatment ( *n* = 19). Univariate analyses were performed with Chi-square testing for categorical variables (Fisher's exact test used for violations of Cochran's assumptions) and Wilcoxon rank-sum test for continuous variables with nonparametric distribution.

**Table 1:** Baseline characteristics of cases before renal infarction overall and divided into treatment groups

	All cases ( *n* = 103)	Anticoagulation ( *n* = 47)	Nonanticoagulation ( *n* = 56)	Antiplatelet ( *n* = 37)
Age (y)	42 (36, 49)	41 (36,49)	44 (36, 48)	46 (39, 50)
Race				
White	46% (47/103)	45% (21/47)	46% (26/56)	49% (18/37)
Black	7% (8/103)	13% (6/47)	4% (2/56)	3% (1/37)
Asian	4% (4/103)	4% (2/56)	4% (2/56)	0% (0/37)
Other	27% (28/103)	21% (10/47)	32 (18/56)	32% (12/37)
Unknown	16% (16/103)	17% (8/47)	14% (8/56)	16% (6/37)
Sex (% male)	80 (82/103)	77 (36/47)	82 (46/56)	86 (32/37)
HTN (%; baseline)	27 (28/103)	23 (11/47)	30 (17/56)	38 (14/37)
New/worsening HTN (% at iRI diagnosis)	49 (50/103)	53 (25/47)	45 (25/56)	51 (19/37)
DM (% baseline)	11 (11/103)	6 (3/47)	14 (8/56)	16 (6/37)
AKI (%at iRI diagnosis)				
SCr 25% rise	9 (8/92)	7 (3/41)	10 (5/51)	6 (2/34)
SCr 50% rise	1 (1/92)	2 (1/41)	0 (0/51)	0 (0/34)
Stage 3 CKD (% eGFR ≤60 mL/min/1.73 m ^2^ at iRI diagnosis)	0 (0/92)	0 (0/40)	0 (0/52)	0 (0/27)
Proteinuria ≥ 1+ (baseline)	1 (1/73)	3 (1/30)	0 (0/43)	0 (0/27)
New Proteinuria ≥ 1+ (% at iRI diagnosis)	9 (8/93)	17 (7/42)	2 a (1/51)	0 b (0/35)
NSAID use (% in year prior to iRI)	77 (79/103)	85 (40/47)	70 (39/56)	70 (26/37)
Long-term followup (median months, IQR)	51.5 (27,99)	48 (22,89)	51.5 (29, 99)	65 (29,114)

Abbreviations: AKI, acute kidney injury; CKD, chronic kidney disease; DM, diabetes mellitus; eGFR, estimated glomerular filtration rate; HTN, hypertension; IQR, interquartile range; iRI, Idiopathic renal infarction; NSAID, nonsteroidal anti-inflammatory drug.

Note: The antiplatelet group is a subgroup of the nonanticoagulation group with clear documentation of treatment.

a *p* = 0.02.

b *p* = 0.01.

## Results

The median follow-up period after iRI was 4.3 years and the background clinical characteristics were similar between treatment groups ( Table 1 ). The overall rates of recurrent arterial and de novo venous thrombosis were 1% (1/103) and 2% (2/103) respectively and there was no significant difference between treatment groups ( Table 2 ). The anticoagulation group had a higher rate of bleeding event than the nonanticoagulation group and the antiplatelet subgroup (13 vs. 0%, *p* = 0.008 and 13 vs. 0%, *p* = 0.03, respectively). Incident proteinuria occurred at iRI in 8% of cases (8/103), 88% (7/8) of which resolved during follow-up. Resolution was also noted in 75% (6/8) patients who presented with acute kidney injury (AKI). Nearly half (50/103) of iRI patients presented with new or worsening hypertension, which resolved in only 18% (9/50) cases. There was no significant difference between treatment groups for long-term resolution of hypertension, proteinuria, or AKI ( Table 2 ). No iRI cases (0/84) had achieved stage 3 CKD and only 2% (2/80) had ≥1+ proteinuria on urinalysis at the last follow-up.

**Table 2:** A comparison of long-term outcomes after idiopathic renal infarction between anticoagulation and nonanticoagulation groups ( *top* ) and between anticoagulation and antiplatelet groups ( *bottom* )

	**Anticoagulation** **(** ***n*** ** = 47)**	**Nonanticoagulation** **(** ***n*** ** = 56)**	***p*** **-Value**
Recurrent arterial thrombosis (% cases)	2 (1/47)	0 (0/56)	0.46
De novo venous thrombosis (%)	4 (2/47)	0 (0/56)	0.21
Bleeding event (%)	13 (6/47)	0 (0/56)	0.008
Resolution of acute hypertension after iRI (%)	16 (4/25)	20 (5/25)	1.0
AKI recovery (%)	100 (3/3)	60 (3/5)	0.46
Stage 3 CKD (% eGFR ≤60 mL/min/1.73 m ^2^ follow-up)	0 (0/30)	0 (0/45)	1.0
SCr > 50% baseline (% last follow-up)	0 (0/39)	2 (1/45)	1.0
Resolution of proteinuria (% negative on UA)	86 (6/7)	100 (1/1)	1.0
Proteinuria ≥ +1 on UA (% follow-up)	6 (2/34)	0 (0/46)	0.18
	**Anticoagulation** **(** ***n*** ** = 47)**	**Antiplatelet** **(** ***n*** ** = 37)**	***p*** **-Value**
Recurrent arterial thrombosis (% cases)	2 (1/47)	0 (0/37)	1.0
Recurrent venous thrombosis (%)	4 (2/47)	0 (0/37)	0.50
Bleeding event (%)	13 (6/47)	0 (0/37)	0.03
Resolution of acute hypertension after iRI (%)	16 (4/25)	26 (5/19)	0.47
AKI recovery (%)	100 (3/3)	50 (1/2)	0.40
Stage III CKD (%) (%eGFR ≤60 mL/min/1.73 m ^2^ last follow-up)	0 (0/39)	0 (0/29)	1.0
SCr > 50% baseline (% last follow-up)	0 (1/39)	3 (1/29)	0.43
Resolution of proteinuria (% negative on UA)	86 (6/7)	NA (0/0)	1.0
Proteinuria ≥ +1 on UA (% last follow-up)	6 (2/34)	0 (0/30)	0.49

Abbreviations: AKI, acute kidney injury; CKD, chronic kidney disease; eGFR, estimated glomerular filtration rate; iRI, Idiopathic renal infarction; UA, urine analysis.

Note: The anticoagulation group included treatment with warfarin, lovenox, rivaroxaban, and apixaban, and the antiplatelet group included aspirin (ASA) and clopidogrel in addition to ASA in one case.

## Discussion

This is the largest retrospective cohort study with the longest follow-up that focuses solely on iRI and the first to compare long-term outcomes between anticoagulation and nonanticoagulation therapy. There was no significant difference in the incidence of recurrent arterial thrombosis or de novo venous thrombosis between treatment groups over 4.3 years median follow-up. In addition there was no significant difference in long-term hypertension, proteinuria, or stage 3 CKD following iRI between treatment groups. There was an increased risk of bleeding events in the anticoagulation group. Most previous studies described renal infarction of all causes. 2 3 4 5 6 7 8 9 10 11 12 13 14 15 All but three studies included less than 10 iRI cases. Oh et al reported a large Asian renal infarction cohort of all etiologies which included 132 iRI cases. 2 Background clinical characteristics and rate of recurrent iRI (2.3%) were similar to our cohort. But the median follow-up for iRI was only 12 months and it did not compare long-term outcomes between anticoagulation and nonanticoagulation treatment groups. Bourgault et al also described a cohort of all types of renal infarction which included 27 iRI cases. 3 Background clinical characteristics were again similar to our cohort but follow-up was limited to 9 months with no evaluation of therapeutic impact on long-term outcomes. Kwon et al compared subgroups with and without AKI and CKD from a cohort of 105 renal infarction cases which included 25 iRI patients. 4 But, there was no separate iRI subgroup analysis.

This study has all the limitations inherent to a retrospective cohort study. There is some ascertainment bias because not all patients had long-term follow-up data for proteinuria, serum creatinine, or blood pressure. While the negative laboratory and imaging studies for secondary causes were directly reviewed for a majority of iRI cases, a minority of cases only had documentation from subspecialists (cardiologists, hematologists, and nephrologists) outside of the military medical system scanned into our EMR which stated that the secondary causes had been ruled out but without all of the specific results. It is possible that there secondary work-up was erroneously incomplete. Unappreciated causes of renal infarction, such as paroxysmal atrial fibrillation, could have confounded our iRI cases. In addition, multivariate analysis for risk of bleeding event was not possible due to the low event rate relative to the number of covariates.

Overall, our data suggest that nonanticoagulation treatment is as efficacious as anticoagulation therapy for prevention of recurrent or de novo thrombosis after iRI, but with less risk of bleeding. In addition, the data suggest that iRI cases should be monitored closely for optimal long-term blood pressure management. A follow-up prospective study would be required to confirm these findings but will be difficult to execute because of disease rarity, case presentation in emergency room departments, and diverse subspecialty follow-up patterns.
